# Improving maintenance of critical care land and aeromedical transfer equipment

**DOI:** 10.1186/cc12221

**Published:** 2013-03-19

**Authors:** D Ashton-Cleary, N Boyd

**Affiliations:** 1 Royal Cornwall Hospital, Truro, UK

## Introduction

The aim was to assess the content and state of repair of equipment carried for transfer of critical care patients to other hospitals. By chance, several items of date-expired stock were identified in the transfer kit whilst moving a patient to a tertiary centre. This raised the possibility of a more extensive problem with the equipment bags. Due to the geographical location of our district general hospital we undertake around 70 transfers of critical care patients to other hospitals per year (16% by air) and it is clearly important that our equipment is well maintained for these journeys.

## Methods

We maintain two identical sets of equipment (syringes, fluid, airway management items, and so forth) and drug bags to take on transfers; one equipment and one drug bag taken on each trip. The contents of all four bags were checked and itemised. By careful consideration of the aims of the bags (to provide emergency equipment and drugs for managing one patient during an *en-route *emergency) a new inventory was devised. Excess items were removed to lighten the bags and improve accessibility to the essential items. Expired stock was removed. A daily checking procedure and tamper-proof seals on the bags were instigated and the bags were reassessed 12 months later.

## Results

A total of 13.9% of drug items and 29.2% of equipment items had expired or would do so within 30 days of the initial assessment. The combined weight of one equipment and one drug bag was reduced from 14 to 9 kg (36% reduction) by introducing the new inventory. At reassessment in November 2012, only 10 items of equipment (3.2%) were expired or near to expiry and there were no expired drug items (4.1% near to expiry). In total, 0.3 kg (26 small items) of extraneous equipment had been added through over-restocking and was removed.

## Conclusion

These bags are designed for a clinician to manage a patient when an emergency arises during transfer of a critical care patient. By the introduction of simple measures, the risks posed by expired items or cluttered equipment bags have almost been eradicated. Significant weight savings have been made; this offers improved ergonomics for staff and is also an important consideration for aeromedical operations. Our department was surprised to discover the extent of decline of our equipment and it may be that other departments would find themselves in a similar position. The anaesthetic registrars who routinely escort the transfer patients have a vested interest to maintain this equipment and this has secured their buy-in to the new checking procedure with clear results.

